# Supplementary material for: Transcriptional Regulation of the Phenylalanine Ammonia-Lyase (PAL) Gene Family in Mulberry Under Chitosan-Induced Stress
Source: Plants (Basel). 2025 Sep 5;14(17):2783. doi: 10.3390/plants14172783 (PMC12430538; doi:10.3390/plants14172783)
Supplement: Supplementary file 1 [file plants-14-02783-s001.zip › Figure S1.pdf]

Figure S1: sequences of the promoter of *PAL* genes in *Morus notabilis*

Figure S1 presents the sequences of the promoter regions for the phenylalanine ammonia-lyase (PAL) gene family in mulberry, encompassing NCBI GeneIDs 21384641, 21407112, 21407113, 21407114, 21407115, and 21409963.

>LOC21384641

5’

CAATCAAGTGAAAAGTGAACTCACCTGCATGGAAACGTAGTTGCTGAAAAATTTCAACTTGAGATTAAA
AGGTGTTAGGTCTTCTTCTTCTGGAGCTCCGTGGAAAAGCCAAAAAGAAGGGCTAAAAAAAAAAAAAA
AAAAAAGGAAGAAGAAGAAGAAGAAAGAGGGACGTGGTTTAAGCGCGCCATTTAATGCTTTCGATGTC
TCATATCAATAGTTTTGGTTTTTCACACATATGCCCCTGAACTTATATGGCTTTAACAACTTACTCCTTAAAC
TTTTAATTTTAATAAGTTGCACTCACAAGTTATAAAATTATTATAATATGTCTCATTTTAACGATAATTGTGTTA
AATTAATGTTAAAAAAATACGTTCAATAAAAAATTTTAACATTCCAATTCAAATGTAATACAAATATTTCAAT
TTTTAAACTTTTAATCCCTTAAGTCTAATAAAAAATTTGAACAAATTTAGTAAAATTGAGTATATTGTA
ACAATTTTACAAGTTCAACAGTTATTAAATTTAAAAGTTTAAGGGGCAATTTACTAAACCATGATAAGTT
CGGGATAATTGTGTTAAAACCACTTTCTTTTATTTGAGATAGTTTGGCCTCGTTCACTTCGCAGATTCGA
GTTTTTAATTCGAGTTGAGACGTGACATTTTCTGAGAGCTTTTCACTGTAGCAGAAAATTTTGATATGAC
ATTTTTTGAGAGCTTTTTACTATAAAAACCTAAAACTGAAAACTCAAATCAGTAAAATGAACGAGACCTT
TATAATAGAAACAGTTATCTTTATTCTACAAGTAATGCTATAGAACATTAATAGTGCTACCACCATCAAC
TAAAAGATGAGTCTTGTGAGACTCATAAATAAAGCTCATTTTTTATTAAATGATGGTGTTTAACCGATGG
TCTCCTTTTAACATTTTTCTTATTCTACCGATGTTTAGGTGATGGTGTTTGGTTGGCGTGAGGCATTCCC
CATCCCTCTGTGGGGCCCACTCCCCCTGTGTGTTGCGGAGTTGTGGATCCACGGACGTGCTTAACTATGAT
CTAATATATATCTTTTGTTTGAATGGATGACATGGTGTAATAAGCAAAAGCAAAAACACATAATTATCA
AATATATATATGTATATATTAGTAAACACATTCTTCCAATAATTAGAGCTCTTGCTATTGGCCATAACGT
TTTTTATATGATATACATTACACAGTTTTATGAGAGCCTTGTGGAAGAACTGTTTCTTTTTTGGCCAAGT
AGTCTCTCTCAAAAGTAAAGAAGAAAAATATTAGTATAAGAATAGGTATGCAGCTCTCATGCACCATAAA
AGATAGAAGGGGGGTCATTGGATTATGTTTTAATGGAAACCGTGATCATATTGTATTCCAAGTCCAAAAT
ATGGCTTTCTTAACAATAAATTTATGGTCGTTAATTAAATACATTAAGAACACATCGAGGAGCTCAAAATAT
TTTCTTGTTTCACAAAATTCTGGGAATAATATAGATGTAGAAGGGAATCATCATGCAATGCCATGAGTGACC
ATCATCTGCCGGCCACATGTCACCTAGCCCTTGGATTGTACGAAGATCCAATAGCCACAAGATACGTAAGG
CTCGTTTAGTTTATTGATTTAAATTTTTAATTCGAGTTGAGATGTGATGTGAGAAATTTTTTACTATAATAAA
AAAATTTAATGTGACTATTCTGAAGAACTTTTTACTGTAACAAAATTCAAATTTTAACTTTAAATGGGTGAA
ATAAATAGTGCCGCAGTCTTTCTTTACAACAAATATCTCCAGCTTGATTATAATCTTTTTTTTTTTCAACACG
TCCAACATAGTAAGCCTTATCTATTTAAAGACCTAGCACTCTATGTAGCTGAACCAATCAACGTCCTCCAA
AAAAAAAAAAAAATAAAACAACTTTCTTGTTACCTTCTTCCTTTCTACATATATAGATCACGCAAACCAAA
GCAAAATACCAATAAAAATC

3’

>LOC21407112

5’

AGAAAAAAGGAATAGTCCTTACCGTCCCCATCAAAACTCAAGCTTTTTTCTTCT
CCAAACTTTATTACCGCACGAGTAATTTGATTAATTTCGACAAAAAAAAGTCACGTCTCTTCATTTAGTT
GCACCAAAACACAATCTTACCTTCTTTTGGTTGGTGGATCCAAAGAAATTAAGTCGATACCCTCCGACCA
CAAATTTTCAGAGTTTTCGCACCAATCAATATATTAGACGTTTCTAAGTACCACTAAGGGCATTTTTATG
ATCATCATGAGACACAAAAAAAAAAAAAAAAAAAAAAAAAAATCCCAACGTACCCCGCTACTTACTTTTAC
CGACCAATATTCATCAAGTTTCCGTCGATTTGCTTACATTTTCCAAAAAAAGATAAAAAAAAAAAAAAGA
AAGAAACTTTACATCAATTAGTTAAAGTTAGGGTTGGCAATTCGTGTTCGCGAGCCGTGTTCGGGCCGAC
ACGATTACGGCATGACACGATTATAGTGAGCACGAACACGACACGATTAATAAACGTGTTAGAAACCCAA
ACACGAACACGACACGCTAATAACACGGTTAACACGACACGACACGCGAACACGATTATTAAACGTGCC
GTGACAAGCCGACACGAAAAAAACACGTTTATGACCCAACTAGCAAAAATAGTGAATTTTATATTGTATTT
AAAACTATATTAATTGACTACTTAATATCACTTATAGTTAAAATCATGCATAATAGTCAACACGCATTAAA
ATATCTAAAACATCACTTATGACATATGTTTCAAAACATACCAATTACTAAACATCTAAAGAAGTTTGAA
AACATAACCAAGTACATTTAAATAAGTTTGAAAACATAACCAAATATTTGAAAATTTAAGTGCATAAAAC
ATAATCAACCATCTACAAGTCCATTGGCATATTTAAGTGCATAAAGCATAAAACATATTTAAAGTTTAGG
TTTATCCATTACGAGTCATCCATTGGCATTTTGTAATCAACCATCTACAAGTCCATCTACAAATCATCCA
TCTACAAGTCCATTGGCATTTTGTAATTTTATCAACATGCCATAACTTATTTAAAAATTAGAGTGTTAAA
TTAAGATAACCCTTTTATCAAACACCCTTCATGCAAATGAGAGAATATAAATAAACTGGGCTGTGAGAGA
ATGAGAGAATGAGAAAGAATGAGAGAATGAGAGAAAGAGAATTAGAGAAGCTAGGGTTTTATCTTTAAA
AAAAATAGAAATATAAATATGGATTAATAAACGGGTCGGTTCGGGTCGTGTCGTGTCGGCCCGATTATGGC
CTGTATAACTAAACGGGTTAAGCGTGTTAAACGGGCTGGCACGAATCCAACCCGTTTATTTTCGTGTTAT
TCGTGTCAGCCCGTTAACGACCCAATTAATAATCGTGCGGTCTAAACCCATTTATTTTGTGTCATTTTTG
TGTCGTGTCTGAAATTGCCGGCCTAGTTAAAGTTACGGTGAGGGTTAATGTTAGTTTTCCACATGTATGT
AATATTTGACTATTAGGTTAGTATTAACTATTAAGTAATGGCATTTACGAAATTTTGTTTTTGCCCTTGC
GATCCCCACCAACCATGCATTTTATTTGACCCTCTCTCTCTATCTCTCCTTCCGTAGCTCCTTCCCACCA
TGCACCCCAGCAGCCACGCAGCCAAATCACAGCCGTTGATCTTTTTCTCTGACATCGAACGACCATTATT
TATATGGTAGCTACTCTCTCTTCCTCTTCTCTTGTGCTCACCTACCAAACCCCAAAGCCATCATTTTTGA
AAACCCCAACTCTCTCTCCTCCTTATTTAAACCCACCATTCTCCTTCCACTTCTCAGGAAATCCGTACAA
CCCATAAAGACCTTTGATTTCCTCTCTCTAAACTCTCTTTCTCTCCCTCTCTCTAGCTCCAACATCTCCA
TATATATATATATATATGTATATATAACACACAACTCTTAAAGCATTTTCCACACA

3’

>LOC21407113

5’

TTTAGCATAAAATAGTAACATTAAAATAAGTTT
ACTTTAATATGTGAGTAGAGTACATTTTATTAATTGTTATACATGATAAACAAAATGAGTGTTACACAAA
TAGTATAAAAGATATTAAAATGAAGAATAAAAAATGTTAAATTTACTGGACCGGTACTGGACCGGAGACA
TCTTCCCAAGGAGAAGAAACGGAAATCACATAGTTATTATAGTTAAGAGTATAGGCATATTGGCATTAAT
GAAATTGTCAGTAAACTTGCATGATTACAAAAAAATGAAAATTAACTCGGTAAGTTGGTGGTGTTTTTTC
GATTATACTGAATTTCACTGTCCAAAAGATTTTTCAATTTCAAGCCCATTGATGATGAGTTTTTGCCCTT
TTTTGGGGTAAAATCCTTCTTTCTTTTTTTTTTTTTGCTCCTTGCCATAGTAAAAAAAAAAAAAAAAAAA
GTAATTCCCTTCTATTTGGCAAAATTTATTGGACTTTCACAGTTCATCAATCGTGCATTAAGCTACAAAT
GACTACTGACTAGACTTCAGTTGAATTCACTCGCGTTCCCACTTTGTTCCATGTGTTGGGCTGGACTTTT
GGGATTGACTTCGGAACAAACTATAGTAAAAATATTGGGCCGTAGAAAGCCACATACGATTAAGCAGGCC
TCTATTTAGAACCAAATCGAAAGGCCCAATCTAGAACCCAACTTAAATAAAGGATAGAACACCTAAAAAA
ATTAAAATAAAGCATAGGGAACAAATGTTCCATTAGCAAAAATAAACAAAAATAAAAATCCCAATTTTTA
TTTTTTTAGTTTTTAAGACGGTTAGTCCTTGAATTATTGCATTTTATTTTTTAGTCTCTGAACTTTTAAT
ATTAACAAATTACTCTTTAAATTTTAATTTTTGACCATTTTAATCCCTACAAACTAAAATTAGCGATGAA
TTTATGAAATTTCATAGAGATCATCACATTTTAAATCTATAATAACTGAAATTTTTACAGAAATAATTAC
ATTAAATATTAAATACTACCTTTATTATTATTATATACCATTAAAAACTAGTTCATTTTGCATTTGATAT
TAGTGTTTTTGTAAAAATTTCAATTACTTTTTCTCCCAGACATGATTATGATCATCTTCGAGAAATTAAA
TAAGTTATTTGTCAATTTTAGTGTGTATTGTGTAGGAACCAAAATGGTTAAAACTAAAAATTTAGAGATT
AATTTATTGAAATTAAAAATATAGAGAACAAAGTGATACAATTGAGATAGTAATAACTTATTACTAAGAG
CATTTTTATGATCATCATGAGACACCAAAAAAAAAAAAAAAAAAAAAAAAAAAAGATCCAAACGTACCCCG
CTACTTACTTTTACCGACAAATATTCGTCAAGTTTCCGTCGATTTGCTTACATTTTCCAAAAAAAAAAAAA
AATTAAAAGAACGAAACTTTACATCAATTAGTTAAAGTTACGGTGAGGGTTAATGTTAGTTTTCCACATG
TATGGAATATTTGACTATTAGGTTAGTATTAACTATTAAGTAATGGCATGTACGAAATTTTGTTTTTGCC
CTTGCAATCCCCACCAACCATGCATTTCCTCTCTCTCTCCTCTCTCTCTCCTCTTTCCTTCCCTAGCTCC
TTCCCACCATGCACCCCAGCAGCCACGCAGCCAAATCACAGCCGTTGATCTTTTTCTCTGACATCGTACG
ACCATTATTTATTTTCAACCAACCCTCCCTTGTGGCTACTCTCTCTTCCTCTTCTCTTGTGCTCACCTAC
CAAACCCCCCCAAAAGCCATAGAGCCATCATCTTTGACAACCCCCAACACTCTCTCCTCCTTATTTAAAC
CCCCACCATTCTCCTTCCACTTCCCAGGAAATCCTTACTACCCATAAAGACCTTTGATTTCCTCTCTCTA
AACTCTCTCTCTCTCTCTAGCTCCAACATCTCCATATTATATATACACACACAACTCTTAAAGCACTTCC
CACACAA

3’

>LOC21407114

5’

CAAGCACGTGCCCTATATTAAA
TGGTCACATTTATTTTCACCATTTTACCTTCTATCATTATTACTTTTTTTTTTTTTGAAGGATCAACCAT
TATTATTTTTTCAATTGTTACAAATTTACAATTACTAATTTTCGTTTCTTTTTCTATTTTGGTTTGTTTC
ACTTTCAGATTCTTGAATGCTGGAATATTTGGCAATGGAACAGAATCATGTCACACACTGCCCCACACAG
CAACAAGATCAGCCCTATTGGTTAGGATCAACACCCTCCTCCAAGGCTACTCAGGCATTAGATTTGAGAT
TTTGGAGGCCATGACAAAGCTTCTCAACAGCAATGTCACCCCGTGCTTGCCGTTGCGCGGCACAGTTACC
GCCTCGGGGGACCTTGTCCCATTGTCCTACATCGCAGGGTTGCTCACCGGCAGGCCTAATTCGAAGGCTG
TGGGCCCCAACGGGGAATCCCTTACCGCCACCCAAGCCTTTAAGGTCGCGGGGATCAACTCGGGGTTCTT
TGAGCTGCAGCCAAAAGAAGGCCTTGCTTTGGTCAATGGTACCGCTGTTGGCTCAGGCTTGGCCTCCATG
GTGCTTTTCGATGCCAACATTCTTTCCGTGTTGTCGGAGATCTTGTCGGCCATTTTCGCGGAAGTCATGC
AGGGTAAGCCCGAGTTCACCGACCACTTGACGCACAAGTTGAAGCACCACCCTGGTCAAATTGAGGCTG
CAGCCATTATGGAACACATTTTGGATGGCAGCTCTTACATGAAGGCTGCAAAGTTGCATGAGATTGACCCT
CTTCAGAAGCCAAAAAAAGACCGCTATGCTCTTAGGACATCACCACAATGGCTTGGACCCCAAATTGAA
GTGATCAGATTCTCAACCAAGTCCATTGAGAGGGAGATCAACTCGGTGAACGACAACCCGTTGATTGAC
GTCTCGAGGAACAAGGCCTTGCACGGCGGCAACTTCCAAGGGACCCCAATTGGAGTTTCCATGGACAAC
ACACGTTTGGCCATTGCCTCAATTGGGAAGCTCATGTTTGCACAATTCTCTGAGCTTGTCAATGACTTTTA
CAACAATGGATTGCCTTCGAACCTCTCCGGTGGTAGGAACCCGAGCTTGGATTATGGCTTTAAAGGCGCT
GAGATTGCCATGGCTTCTTATTGCTCTGAGCTCCAATCCCTTGCCAACCCTGTCACTAGCCATGTCCAAAG
CGCTGAGCAACACAACCAAGATGTGAACTCTTTGGGATTGATCTCTTCGCGCAAAACCGCGGAGTCAATT
GACATTCTCAAGCTCATGTCCACCACATTCTTGGTGGGTCTATGCCAAGCCATTGACTTGAGGCACTTGGA
GGAGAATTTGAAGCACACGGTTAAGAACACCGTGAGCCAAGTGGTGAAGAGAGTGCTAACAACTGGCG
TAAACGGAGAGCTTAACCCGTCGAGGTTCTGCGAGAAGGATCTCCTCAAAGTGGTGGACAGGGAATATG
TCTATACCTACATTGATGACCCCTGCAGTGCTACCTACCCGTTGATGCAAACATTGAGGCAAGTACTCGTC
GAGCACGCGCTGACAAACGGTGAAAGCGAGAAGAACACGAGCACCTCAATCTTCCAAAAGATTGCAGC
CTTTGAGGAGGAGCTGAAATCCCTTTTGCCTAAGGAGGTGGAGAGCGCAAGGATCGCTTATGAGAACGG
GAACGCCGCGATTTCGAACAAGATCACCGAGTGCCGGTCTTACCCCTTGTACAAGTTTGTGAGGGAGGA
GTTGGGGACTAGTTTGCTAACAGGTGAGAAAGTTAGATCACCAGGGGAGGAATTTGACAAGGTTTATACT
GCCATGTGCCAAGGCAAGCTTATTGATCCAATGTTGGAGTGCCTTAGTGGCTGGAATGGTGAACCTCTTCC
AATCTGTTAGTGTTGTTTTTTTTTTATTCCTTTTTTTCTTTTGGGGCTGAATTTGAATGTTGGTCTTAATCATA
TTTGTATGTGTTCTTAAAA

3’

>LOC21407115

5’

CGTCTACACCCCAAATAGGAGAGAAATTAAAATTAAAAAAAAAAAAAAAAAAAACTGTTTGGGTCATCGT
AGACAACGTATTTAGAGATGCCTGGTGGTCTCTTTCATTAAAAACAGAAAATTCAGAGGGTCGAAATTCT
CCACTAGGACCACCTCACGGTAAGAGAGTAGTTTAACCTTAAGATTTAAATTGGACGGCGCTTATGATAGA
GTGCGACAGTACAGTGCAATCACTTAATTTTGAAGAATCCAAATTTCAATACAATACTAGCCGTCGAATGT
GACGAATGGCCCTGTGAGGAGCCGTCGAATGTGACGAATGACTCTGTGAGATTTATGGTGAGCGTGAAC
AAAGCTAAATAAGGTTTAATGGACGCTTAAACCCTTACTCAACTAATTTTTTTCTTAAAAATATATGTATATA
AATATGCTCATACTCTCTATGAAACATTAAAATTTAATCTTAAAATCTTATTATTATTATTATTATTATTAATAAC
CCCATTGATTGAAGTTTATGGCTTCATATAACATTAATACTTAATCTTATATTCTTACTATTATTGTTTTAATT
ATTAACCCCATTGATAGAAGTTTATGGCTTCATATATATATATATATCATTAAGATTTAATCTTATATTC
TTATCATTGTTTTAATTATTAATCTCATTGATAGAAGTTTATGGCTTCATATATAACTGATAATGAGGGA
ATGCACCCACCATAAAATGCCACTTCTTTCCCCTCTTTAAAAAAAAAAAAAAAAAGAAAAAGAAAAAGAA
AAGTGACATGCCACTTGAATATTATTAGTCACTATCTCTTCCTTGTCAGTTTTCCGCACATGGCGCAATGT
GAAAAATATTGGGAATAAAAAAAAATAAAGAAAAAAGGAATAGTCCTTACCCTCCCCATCAAAACACAAG
CTTTTTTCTTCACCAAACTTTATTACCGCACGAGTAATTTGATTAATTTTGACCAAAAAAAAGTCACGTCA
CTTCAATTACTTGCATCAAAACACAATCTTACCTGCTTGTTGGTTGGCGGATCCAAAGAAATTAAGTGGA
TACCCTCGGACCACAAATTTTCAGAGTTTTCACACCAATCAATATATTACACGTTTCTAAGTACCACTAA
GAGCATTTTTATGATCTACATGTGACACCAAAAAAAAAAAAAAAAAAAAAAAAATCCCAACGTACCCCGCT
ACTTACTTTTACCGTCAAATATTCATCAAGTTTCCGTCCATTTACTTACATTTTCAAAAAAAAAAAAAAAA
AAAAAGGAACTTTACATCAATTAGTTAAAAGTTACGGTGAGGGTTAATGTTAGTTTTCCACATGTATGGA
ATATTTGACTATTAGGTTAGTATTAACTATTAAGGCCTCGTTTATTTTGTTGATTCGAGTTTAGAATTTG
AGTTTTATTACAGTAAAAAGCTCTCAGAAACAGTCATATCTAACTTTTTTGCTACAGTAAAAAGTTCTCA
TATAAAATCACATCTCATCTCAAATTACAAACTCAAATCAACGAAATGAACAAGATCTAAGTAATGGCAT
TTAAGAAATTTTGTTTTTGCCCTTGCAATCCCCACCAACCATGCATTTCATTTCACCCCCTCTCTCTCTC
CTCTCTCTCTCCTAGCTCCTTCCCACCATGCACCCCAGCAGCCACGCAGCCAAATCACAGCCGTTGATCT
TTTTCTCTGACATCGTACGACCATTATTTATTTTCAACCAACCCACCCTTGTGGCTACTCTCTCTTCCTC
TTCTCTTGTGCTCACCTACCAAACCCCCCCAAAAGCCATAGAGCCATCATCTTTGACAACCCCCAACACT
CTCTCCTCCTTATTTAAACCCCCACCATTCTCCTTCCACTTCCCAGGAAATCCTTACTACCCATAAAGAC
CTTTGATTTCCTCTCTCTAAACTCTCTCTCTCTCTCTCTAGCTCCAACATCTCCATATATATATACACAC
ACAACTCTTAAAGCCCTTCCCACACAA

3’

>LOC21409963

5’

AATAATTTAAAAGCGATGCCAAAGGATGAAGAGGAAGCTTTAGATGAGGAAGATTATGAGGAACATGGA
GATACCTTGTGTGGTGCATGTGGGAAGAACTATGCTGCTGATGAGTTCTGGATTTGTTGCGACATATGTGA
GAAGTGGTTTCATGGGAAGTGTGTGAAGATCACTCCCGCCAGAGCTCAGCACATTAAGCAGTACAAGTG
CCCTTCATGCAGCAACAAGAGAGCACGTGTTTGATATCACAAGCAAGGCGGGTTAGTCCTAGCGATCGTA
TTCGTGTCATGCCCGCTCCAGCTAACTGTTTAGTCGCTCCATATTGAGCCCATGTTGGCTCGGCATTTGCAC
TCAACACTTTCTTAAGAAACTCTTTTCGTTTGTAGTGTTTAGGCTCTGAACATGTGTTGCATAGGATTGTAA
TTTGGTAAGTATCTCTTGCATTGTTATAGCATTTGTTCTCTTCAATGAGAAAGGCACTGATTATCGTCATCCC
ATTTATATTTACGTTTTTGAGTTCATCGCAAACATGTTATGATCTGTTTTGGGAGAGAGAAGGGAACGGTTT
TGACTTTCGATGTCCCCGAAACTCTTAGAAACCAATCCGTATCTACAGATTTTCTAGAAGTCGAAGTCTAC
GCAGAAATGCATTTCTAAATTCTGTCAATGCTTCAAAGTCGACACTAGTAAGAAGTGGAAGTGCAACCTC
CACGCTATAGGAGGATGAAACTAGTAAAAATAGTTTCTCCACGCGATAGATGTGTTGGTTTTTCCAACTTC
CAGTGATTTGGACTAGTTTTTTTTTTTTTTTTTTTTTTTGGTACGTCTAATGTGTTTACTGTTGCATGGTTTTG
ATCCTTGAAAAGTGAGAAGTGGTTTCATGGGAAGTGTGTGACCGCAACGTTTAGCCCCTCTTTTTAAGAT
TTTATTTTGTCTTTCTCCTCGTAATGATCTTTACTTTGTAACCATGGAAATAATGTGTATTGCTTATGAGAACA
CGTGTGGTTCATTTTATTCATCAGAGATGTATGACGACAAAAATACACATTAGTTGTTGTCCATAAGTTCAA
TTGCATACGTCGTTTATATATTATGCCCAGGATATGCAAAACTCCATTGCATCTTTCAATTGCAAAATTGGGT
TCTCGCTTTCTTGGCTTTCCAATAGGATATTGCGCTATCATCCCATGTTTCAATGAGCATTAAATGAAGTTTC
TCTTGCTCTCGCAAGCCAGGACACTTGGTCAAAGTTAGTGTTATACACGATGGTGAGCTAGCTAAAAGAC
AGCAGAAGTTTAGTTAACATGAAAAGGCATGAAACTTGAAACACATTTACATGCATTATTTGGTCACATTT
GGTGCGCATTTAATGTTGGTTAGTTAAAAGACATCATTAGTCGGTAAGTTGATTTAAAAACGGGCCGGATA
TGAAATACATGTCAATTCATTTTAAATCCTATCTGTAGATTCCATTTTAAATCGTTTAATCAGTTTTGCATACT
TTTTCGCTATAACGTGATTTTTGATCAAATTTAATGTTCAAAGTACTCTTTAATTATGTTCCCACGAAAGA
ATGAAAAAGAAAAAACCACGAGAAAGGTTGTTGGCTGCGAGTAGTGTCATTTTCCACTTGGTCAATGGT
CGGACACTAAGAAAAAGGTAAATGAGACCATACACAGTAACATGCACAAACAAAATCTTCTCCTAAGTA
AATGCCATAACAACTTCCAGTGCTTTTCCATCTAACTCCAAATCCTCTTTTTTCTTTCTACAAAAGACTTGA
CTAAACCCTTTGAAGCAGCCATTTCCCACTTCTACTGGCTCTCATAAACTCATTTCACTCCTATAAATTC
AATCCACTTCCACTTCTTTCCCTCCCACACTCAAAAACACTTCCAAAAACTTTAAACCCAAATATCATCA
TCCCAACCATTTTACCAAGAACTTCAAAAACTTCCATCATCCCAATTCCCAAACATTCCTTTTTCCAAAA
TCCAAAA

3’
