# Supplementary material for: Transcriptional Regulation of the Phenylalanine Ammonia-Lyase (PAL) Gene Family in Mulberry Under Chitosan-Induced Stress
Source: Plants (Basel). 2025 Sep 5;14(17):2783. doi: 10.3390/plants14172783 (PMC12430538; doi:10.3390/plants14172783)
Supplement: Supplementary file 1 [file plants-14-02783-s001.zip › Table S1.pdf]

Table S1: Genomic analysis of plant regulatory motifs and their transcription factor families via PlantRegMap

Motif represents a DNA sequence pattern that transcription factors recognize and bind to, typically shown as a consensus sequence or position weight matrix.

Family indicates the classification group of transcription factors that share similar DNA-binding domain structures, such as C2H2 zinc finger, homeodomain, or bHLH families.

Seq ID and **Position** identify the specific location where a motif is found in the genomic sequence, with Position showing the exact nucleotide coordinates.

Strand indicates whether the motif is found on the positive (+) or negative (-) DNA strand.

P-value and **Q-value** are statistical measures of significance - the p-value represents the probability of finding a match by chance, while the q-value is an adjusted p-value that accounts for multiple testing.

Matched sequence shows the actual DNA sequence that matches the predicted motif pattern in the genome.

>LOC21384641

Motif	Family	Seq ID	Position	Strand	p-value	q-value	Matched sequence
L484_005589	AP2	LOC21384641	107-126	+	2.36e-06	0.000762	CAAAAAAAAAAAAAAATAAA
L484_005589	AP2	LOC21384641	108-127	+	6.16e-06	0.000993	AAAAAAAAAAAAAAATAAAA
L484_005589	AP2	LOC21384641	109-128	+	3.05e-05	0.00248	AAAAAAAAAAAAAATAAAAC
L484_005589	AP2	LOC21384641	112-131	+	3.08e-05	0.00248	AAAAAAAAAAATAAAACAAC
L484_005589	AP2	LOC21384641	106-125	+	6.25e-05	0.00403	CCAAAAAAAAAAAAAAAATAA
L484_026782	AP2	LOC21384641	167-181	-	8.54e-05	0.0318	CTTTGGTTTGCGTGA
L484_019400	BBR-BPC	LOC21384641	142-162	+	6.87e-05	0.0217	CCTTCTTCCTTTCTACATATA
L484_025208	Dof	LOC21384641	106-126	-	7.65e-06	0.00236	TTTATTTTTTTTTTTTTTTGG
L484_025208	Dof	LOC21384641	111-131	-	1.76e-05	0.00272	GTTGTTTTATTTTTTTTTTTT
L484_021308	Dof	LOC21384641	135-155	-	3.77e-05	0.0122	AGAAAGGAAGAAGGTAACAAG
L484_025208	Dof	LOC21384641	105-125	-	9.5e-05	0.00979	TTATTTTTTTTTTTTTTTGGA

Motif	Family	Seq ID	Position	Strand	p-value	q-value	Matched sequence
L484_027201	GRAS	LOC21384641	107-126	+	7.67e-05	0.0244	CAAAAAAAAAAAAAAAATAAA
L484_023454	HD-ZIP	LOC21384641	92-101	-	8.59e-05	0.0319	CGTTGATTGG
L484_003817	MIKC_MADS	LOC21384641	106-119	+	5.68e-05	0.0193	CCAAAAAAAAAAAA
L484_019417	MIKC_MADS	LOC21384641	104-118	+	9.27e-05	0.0318	CTCCAAAAAAAAAAA
L484_018377	MYB	LOC21384641	52-61	-	7.7e-06	0.00281	TAGATAAGGC
L484_023094	MYB	LOC21384641	53-65	-	8.56e-06	0.00307	TAAATAGATAAGG
L484_026360	MYB	LOC21384641	51-64	+	4.26e-05	0.0156	AGCCTTATCTATTT
L484_019058	MYB	LOC21384641	48-62	+	6.07e-05	0.0194	GTAAGCCTTATCTAT
L484_027259	MYB_related	LOC21384641	51-65	+	8.76e-06	0.00304	AGCCTTATCTATTTA
L484_024069	MYB_related	LOC21384641	49-63	+	2.87e-05	0.0102	TAAGCCTTATCTATT
L484_017072	MYB_related	LOC21384641	51-65	-	6.3e-05	0.0216	TAAATAGATAAGGCT
L484_013470	MYB_related	LOC21384641	67-76	+	8.89e-05	0.0329	AGACCTAGCA
L484_022972	RAV	LOC21384641	40-56	-	4.77e-05	0.0166	AAGGCTTACTATGTTGG
L484_007070	WOX	LOC21384641	90-99	+	7.01e-05	0.0251	AACCAATCAA

>LOC21407112

Motif	Family	Seq ID	Position	Strand	p-value	q-value	Matched sequence
L484_005589	AP2	LOC21407112	110-129	-	2.88e-06	0.000914	TAGAGAGAGGGAGAGAAAGA
L484_005589	AP2	LOC21407112	112-131	-	6.21e-06	0.000987	GCTAGAGAGAGGGAGAGAAA
L484_005589	AP2	LOC21407112	108-127	-	1.25e-05	0.00104	GAGAGAGGGAGAGAAAGAGA
L484_005589	AP2	LOC21407112	106-125	-	1.31e-05	0.00104	GAGAGGGAGAGAAAGAGAGT
L484_005589	AP2	LOC21407112	15-34	-	1.94e-05	0.00123	TTAAATAAGGAGGAGAGAGA
L484_019400	BBR-BPC	LOC21407112	107-127	+	1.06e-10	3.16e-08	CTCTCTTTCTCTCCCTCTCTC
L484_019276	BBR-BPC	LOC21407112	104-127	-	4.77e-10	1.31e-07	GAGAGAGGGAGAGAAAGAGAGTTT
L484_019276	BBR-BPC	LOC21407112	106-129	-	2.04e-09	2.46e-07	TAGAGAGAGGGAGAGAAAGAGAGT
L484_019276	BBR-BPC	LOC21407112	108-131	-	2.69e-09	2.46e-07	GCTAGAGAGAGGGAGAGAAAGAGA
L484_019400	BBR-BPC	LOC21407112	105-125	+	4.71e-09	7.03e-07	AACTCTCTTTCTCTCCCTCTC
L484_019400	BBR-BPC	LOC21407112	109-129	+	8.16e-09	8.13e-07	CTCTTTCTCTCCCTCTCTCTA
L484_019276	BBR-BPC	LOC21407112	110-133	-	2.53e-08	1.74e-06	GAGCTAGAGAGAGGGAGAGAAAGA
L484_019400	BBR-BPC	LOC21407112	103-123	+	5.94e-08	4.44e-06	TAAACTCTCTTTCTCTCCCTC
L484_019276	BBR-BPC	LOC21407112	102-125	-	1.21e-07	6.64e-06	GAGAGGGAGAGAAAGAGAGTTTAG
L484_019400	BBR-BPC	LOC21407112	111-131	+	1.52e-07	7.68e-06	CTTTCTCTCCCTCTCTCTAGC
L484_019400	BBR-BPC	LOC21407112	113-133	+	1.54e-07	7.68e-06	TTCTCTCCCTCTCTCTAGCTC
L484_019276	BBR-BPC	LOC21407112	114-137	-	2.34e-07	1.07e-05	GTTGGAGCTAGAGAGAGGGAGAGA
L484_019276	BBR-BPC	LOC21407112	112-135	-	1.38e-06	5.43e-05	TGGAGCTAGAGAGAGGGAGAGAAA
L484_019400	BBR-BPC	LOC21407112	115-135	+	1.84e-06	7.84e-05	CTCTCCCTCTCTCTAGCTCCA
L484_019276	BBR-BPC	LOC21407112	100-123	-	5.7e-06	0.000176	GAGGGAGAGAAAGAGAGTTTAGAG
L484_019276	BBR-BPC	LOC21407112	116-139	-	5.77e-06	0.000176	ATGTTGGAGCTAGAGAGAGGGAGA
L484_019400	BBR-BPC	LOC21407112	8-28	+	8.02e-06	0.0003	CCCCAACTCTCTCTCCTCCTT

Motif	Family	Seq ID	Position	Strand	p-value	q-value	Matched sequence
L484_019400	BBR-BPC	LOC21407112	117-137	+	3.07e-05	0.00102	CTCCCTCTCTCTAGCTCCAAC
L484_019276	BBR-BPC	LOC21407112	7-30	-	5.08e-05	0.0014	ATAAGGAGGAGAGAGAGTTGGGGT
L484_019400	BBR-BPC	LOC21407112	10-30	+	6.51e-05	0.00195	CCAACTCTCTCTCCTCCTTAT
L484_019400	BBR-BPC	LOC21407112	101-121	+	9.26e-05	0.00252	TCTAAACTCTCTTTCTCTCCC
L484_025451	C2H2	LOC21407112	10-28	+	1.43e-05	0.00306	CCAACTCTCTCTCCTCCTT
L484_025451	C2H2	LOC21407112	41-59	+	2.07e-05	0.00306	CATTCTCCTTCCACTTCTC
L484_025451	C2H2	LOC21407112	13-31	+	3.32e-05	0.00328	ACTCTCTCTCCTCCTTATT
L484_025451	C2H2	LOC21407112	108-126	+	7.48e-05	0.00491	TCTCTTTCTCTCCCTCTCT
L484_008190	C2H2	LOC21407112	105-116	-	8.01e-05	0.0286	AGAAAGAGAGTT
L484_025451	C2H2	LOC21407112	111-129	+	8.29e-05	0.00491	CTTTCTCTCCCTCTCTCTA
L484_024949	GATA	LOC21407112	131-145	-	7.87e-06	0.00287	ATGGAGATGTTGGAG
L484_027201	GRAS	LOC21407112	106-125	-	3.49e-07	0.000103	GAGAGGGAGAGAAAGAGAGT
L484_027201	GRAS	LOC21407112	108-127	-	6.27e-07	0.000103	GAGAGAGGGAGAGAAAGAGA
L484_027201	GRAS	LOC21407112	9-28	-	1.43e-05	0.00156	AAGGAGGAGAGAGAGTTGGG
L484_010123	SBP	LOC21407112	65-75	-	2.99e-06	0.00105	GTTGTACGGAT
L484_024784	SBP	LOC21407112	66-76	+	5.64e-06	0.00198	TCCGTACAACC
L484_018032	SBP	LOC21407112	64-77	-	9.21e-06	0.00338	GGGTTGTACGGATT
L484_014488	SBP	LOC21407112	66-73	+	1.11e-05	0.00396	TCCGTACA
L484_013868	SBP	LOC21407112	66-74	-	5.24e-05	0.0174	TTGTACGGA
L484_010322	SBP	LOC21407112	65-74	-	7.51e-05	0.0246	TTGTACGGAT
L484_011281	SBP	LOC21407112	67-76	+	7.86e-05	0.0261	CCGTACAACC

>LOC21407113

Motif	Family	Seq ID	Position	Strand	p-value	q-value	Matched sequence
L484_005589	AP2	LOC21407113	129-148	-	1.79e-05	0.00601	TGGAGCTAGAGAGAGAGAGA
L484_005589	AP2	LOC21407113	125-144	-	5.65e-05	0.00946	GCTAGAGAGAGAGAGAGAGT
L484_019400	BBR-BPC	LOC21407113	122-142	+	3.36e-09	7.53e-07	TAAACTCTCTCTCTCTCTCTA
L484_019400	BBR-BPC	LOC21407113	126-146	+	5.47e-09	7.53e-07	CTCTCTCTCTCTCTCTAGCTC
L484_019400	BBR-BPC	LOC21407113	124-144	+	7.57e-09	7.53e-07	AACTCTCTCTCTCTCTCTAGC
L484_019276	BBR-BPC	LOC21407113	127-150	-	2.33e-08	6.44e-06	GTTGGAGCTAGAGAGAGAGAGAGA
L484_019400	BBR-BPC	LOC21407113	120-140	+	1.44e-07	1.07e-05	TCTAAACTCTCTCTCTCTCTC
L484_019400	BBR-BPC	LOC21407113	128-148	+	2.07e-07	1.24e-05	CTCTCTCTCTCTCTAGCTCCA
L484_019276	BBR-BPC	LOC21407113	123-146	-	3.3e-07	3.97e-05	GAGCTAGAGAGAGAGAGAGAGTTT
L484_019276	BBR-BPC	LOC21407113	125-148	-	4.32e-07	3.97e-05	TGGAGCTAGAGAGAGAGAGAGAGT
L484_019276	BBR-BPC	LOC21407113	121-144	-	7.48e-07	5.16e-05	GCTAGAGAGAGAGAGAGAGTTTAG
L484_019276	BBR-BPC	LOC21407113	119-142	-	1.42e-06	7.86e-05	TAGAGAGAGAGAGAGAGTTTAGAG
L484_019276	BBR-BPC	LOC21407113	129-152	-	1.9e-06	8.75e-05	ATGTTGGAGCTAGAGAGAGAGAGA
L484_019400	BBR-BPC	LOC21407113	130-150	+	9.81e-06	0.000488	CTCTCTCTCTCTAGCTCCAAC
L484_019276	BBR-BPC	LOC21407113	117-140	-	2.88e-05	0.00114	GAGAGAGAGAGAGAGTTTAGAGAG
L484_019276	BBR-BPC	LOC21407113	113-136	-	4.41e-05	0.00152	GAGAGAGAGAGTTTAGAGAGAGGA
L484_019400	BBR-BPC	LOC21407113	25-45	+	5.69e-05	0.00242	CCCCAACACTCTCTCCTCCTT
L484_019400	BBR-BPC	LOC21407113	118-138	+	8.48e-05	0.00316	TCTCTAAACTCTCTCTCTCTC
L484_017006	C2H2	LOC21407113	25-35	-	1.74e-05	0.00618	GAGTGTTGGGG
L484_025451	C2H2	LOC21407113	24-42	+	5.27e-05	0.00649	CCCCCAACACTCTCTCCTC
L484_025451	C2H2	LOC21407113	54-72	+	6.78e-05	0.00649	CCCCACCATTCTCCTTCCA
L484_025451	C2H2	LOC21407113	27-45	+	7.3e-05	0.00649	CCAACACTCTCTCCTCCTT
L484_025451	C2H2	LOC21407113	60-78	+	9.91e-05	0.00661	CATTCTCCTTCCACTTCCC

Motif	Family	Seq ID	Position	Strand	p-value	q-value	Matched sequence
L484_011629	ERF	LOC21407113	23-43	+	7.12e-05	0.0232	ACCCCCAACACTCTCTCCTCC
L484_024949	GATA	LOC21407113	144-158	-	7.87e-06	0.00274	ATGGAGATGTTGGAG
L484_014769	GATA	LOC21407113	9-27	+	3.9e-05	0.0135	CATCATCTTTGACAACCCC
L484_027201	GRAS	LOC21407113	121-140	-	1.61e-05	0.00541	GAGAGAGAGAGAGAGTTTAG
L484_005010	MYB	LOC21407113	17-31	+	8e-05	0.0247	TTGACAACCCCCAAC

>LOC21407114

Motif	Family	Seq ID	Position	Strand	p-value	q-value	Matched sequence
L484_005589	AP2	LOC21407114	128-147	-	2.13e-06	0.000731	AAGAAAAAAAGGAATAAAAA
L484_005589	AP2	LOC21407114	123-142	-	6.04e-06	0.000904	AAAAAGGAATAAAAAAAAAA
L484_005589	AP2	LOC21407114	121-140	-	8.77e-06	0.000904	AAAGGAATAAAAAAAAAACA
L484_005589	AP2	LOC21407114	129-148	-	1.28e-05	0.000904	AAAGAAAAAAAGGAATAAAA
L484_005589	AP2	LOC21407114	130-149	-	1.31e-05	0.000904	AAAAGAAAAAAAGGAATAAA
L484_005589	AP2	LOC21407114	125-144	-	1.81e-05	0.00104	AAAAAAAGGAATAAAAAAAA
L484_005589	AP2	LOC21407114	116-135	-	3.8e-05	0.00187	AATAAAAAAAAAACAACACT
L484_005589	AP2	LOC21407114	131-150	-	5.73e-05	0.00246	CAAAAGAAAAAAAGGAATAA
L484_005589	AP2	LOC21407114	119-138	-	7.17e-05	0.00274	AGGAATAAAAAAAAAACAAC
L484_019276	BBR-BPC	LOC21407114	126-149	-	7.78e-05	0.0136	AAAAGAAAAAAAGGAATAAAAAAA
L484_019276	BBR-BPC	LOC21407114	124-147	-	8.04e-05	0.0136	AAGAAAAAAAGGAATAAAAAAAAA
L484_022662	bHLH	LOC21407114	36-45	+	4.96e-05	0.0187	TGCCATGTGC
L484_025451	C2H2	LOC21407114	10-28	-	3.88e-05	0.0135	CCTTGTCAAATTCCTCCCC
L484_001930	CPP	LOC21407114	157-166	+	2.2e-05	0.00836	AATTTGAATG
L484_021308	Dof	LOC21407114	123-143	-	2.09e-07	7.29e-05	AAAAAAGGAATAAAAAAAAAA
L484_014436	Dof	LOC21407114	122-142	+	6.8e-07	0.000225	GTTTTTTTTTTATTCCTTTTT
L484_008109	Dof	LOC21407114	128-148	-	9.71e-07	0.000335	AAAGAAAAAAAGGAATAAAAA
L484_014436	Dof	LOC21407114	130-150	+	1.44e-06	0.000238	TTTATTCCTTTTTTTCTTTTG
L484_021308	Dof	LOC21407114	131-151	-	2.74e-06	0.000477	CCAAAAGAAAAAAAGGAATAA
L484_005785	Dof	LOC21407114	131-151	-	3.42e-06	0.00116	CCAAAAGAAAAAAAGGAATAA
L484_008599	Dof	LOC21407114	131-149	-	4.71e-06	0.00166	AAAAGAAAAAAAGGAATAA
L484_021308	Dof	LOC21407114	122-142	-	8.71e-06	0.00101	AAAAAGGAATAAAAAAAAAAC
L484_015022	Dof	LOC21407114	131-151	-	1.13e-05	0.00393	CCAAAAGAAAAAAAGGAATAA
L484_025208	Dof	LOC21407114	123-143	+	1.15e-05	0.00178	TTTTTTTTTTATTCCTTTTTT
L484_025208	Dof	LOC21407114	124-144	+	1.53e-05	0.00178	TTTTTTTTTATTCCTTTTTTT
L484_025208	Dof	LOC21407114	125-145	+	1.59e-05	0.00178	TTTTTTTTATTCCTTTTTTTC
L484_025208	Dof	LOC21407114	122-142	+	2.44e-05	0.00206	GTTTTTTTTTTATTCCTTTTT
L484_016668	Dof	LOC21407114	130-143	-	2.78e-05	0.0102	AAAAAAGGAATAAA
L484_008109	Dof	LOC21407114	136-156	-	5.85e-05	0.0101	CAGCCCCAAAAGAAAAAAAGG

Motif	Family	Seq ID	Position	Strand	p-value	q-value	Matched sequence
L484_025208	Dof	LOC21407114	133-153	+	6.03e-05	0.00359	ATTCCTTTTTTTCTTTTGGGG
L484_025208	Dof	LOC21407114	119-139	+	6.39e-05	0.00359	GTTGTTTTTTTTTTATTCCTT
L484_009894	G2-like	LOC21407114	126-140	+	6.53e-05	0.024	TTTTTTTATTCCTTT
L484_024949	GATA	LOC21407114	83-97	+	9.76e-05	0.0357	GTGGCTGGAATGGTG
L484_003817	MIKC_MADS	LOC21407114	138-151	-	4.21e-05	0.0148	CCAAAAGAAAAAAA
L484_027583	Trihelix	LOC21407114	94-101	+	6.17e-05	0.0117	GGTGAACC
L484_027583	Trihelix	LOC21407114	94-101	-	6.17e-05	0.0117	GGTTCACC
L484_026456	YABBY	LOC21407114	174-181	-	6.07e-05	0.0223	TATGATTA
L484_014588	ZF-HD	LOC21407114	170-184	-	7.17e-05	0.0267	AAATATGATTAAGAC

>LOC21407115

Motif	Family	Seq ID	Position	Strand	p-value	q-value	Matched sequence
L484_005589	AP2	LOC21407115	126-145	-	4.68e-06	0.00122	GCTAGAGAGAGAGAGAGAGA
L484_005589	AP2	LOC21407115	124-143	-	7.33e-06	0.00122	TAGAGAGAGAGAGAGAGAGT
L484_005589	AP2	LOC21407115	130-149	-	1.79e-05	0.002	TGGAGCTAGAGAGAGAGAGA
L484_019400	BBR-BPC	LOC21407115	123-143	+	3.42e-11	1.02e-08	AACTCTCTCTCTCTCTCTCTA
L484_019400	BBR-BPC	LOC21407115	125-145	+	2.15e-10	3.19e-08	CTCTCTCTCTCTCTCTCTAGC
L484_019400	BBR-BPC	LOC21407115	121-141	+	1.06e-09	1.05e-07	TAAACTCTCTCTCTCTCTCTC
L484_019276	BBR-BPC	LOC21407115	122-145	-	4.92e-09	1.2e-06	GCTAGAGAGAGAGAGAGAGAGTTT
L484_019400	BBR-BPC	LOC21407115	127-147	+	5.47e-09	4.06e-07	CTCTCTCTCTCTCTCTAGCTC
L484_019276	BBR-BPC	LOC21407115	124-147	-	8.86e-09	1.2e-06	GAGCTAGAGAGAGAGAGAGAGAGT
L484_019276	BBR-BPC	LOC21407115	128-151	-	2.33e-08	2.11e-06	GTTGGAGCTAGAGAGAGAGAGAGA
L484_019276	BBR-BPC	LOC21407115	120-143	-	7.03e-08	4.78e-06	TAGAGAGAGAGAGAGAGAGTTTAG
L484_019276	BBR-BPC	LOC21407115	126-149	-	9.26e-08	5.03e-06	TGGAGCTAGAGAGAGAGAGAGAGA
L484_019400	BBR-BPC	LOC21407115	119-139	+	1.44e-07	8.55e-06	TCTAAACTCTCTCTCTCTCTC
L484_019276	BBR-BPC	LOC21407115	118-141	-	1.5e-07	6.81e-06	GAGAGAGAGAGAGAGAGTTTAGAG
L484_019400	BBR-BPC	LOC21407115	129-149	+	2.07e-07	1.03e-05	CTCTCTCTCTCTCTAGCTCCA
L484_019276	BBR-BPC	LOC21407115	130-153	-	1.9e-06	7.38e-05	ATGTTGGAGCTAGAGAGAGAGAGA
L484_019400	BBR-BPC	LOC21407115	131-151	+	9.81e-06	0.000416	CTCTCTCTCTCTAGCTCCAAC
L484_019276	BBR-BPC	LOC21407115	116-139	-	2.88e-05	0.000979	GAGAGAGAGAGAGAGTTTAGAGAG
L484_019276	BBR-BPC	LOC21407115	112-135	-	4.41e-05	0.00133	GAGAGAGAGAGTTTAGAGAGAGGA
L484_019400	BBR-BPC	LOC21407115	24-44	+	5.69e-05	0.00211	CCCCAACACTCTCTCCTCCTT
L484_019400	BBR-BPC	LOC21407115	117-137	+	8.48e-05	0.0028	TCTCTAAACTCTCTCTCTCTC
L484_017006	C2H2	LOC21407115	24-34	-	1.74e-05	0.00618	GAGTGTTGGGG

Motif	Family	Seq ID	Position	Strand	p-value	q-value	Matched sequence
L484_025451	C2H2	LOC21407115	23-41	+	5.27e-05	0.00623	CCCCCAACACTCTCTCCTC
L484_025451	C2H2	LOC21407115	53-71	+	6.78e-05	0.00623	CCCCACCATTCTCCTTCCA
L484_025451	C2H2	LOC21407115	26-44	+	7.3e-05	0.00623	CCAACACTCTCTCCTCCTT
L484_025451	C2H2	LOC21407115	59-77	+	9.91e-05	0.00635	CATTCTCCTTCCACTTCCC
L484_011629	ERF	LOC21407115	22-42	+	7.12e-05	0.0231	ACCCCCAACACTCTCTCCTCC
L484_024949	GATA	LOC21407115	145-159	-	7.87e-06	0.00275	ATGGAGATGTTGGAG
L484_014769	GATA	LOC21407115	8-26	+	3.9e-05	0.0136	CATCATCTTTGACAACCCC
L484_027201	GRAS	LOC21407115	124-143	-	5.34e-06	0.0018	TAGAGAGAGAGAGAGAGAGT
L484_027201	GRAS	LOC21407115	120-139	-	1.61e-05	0.0027	GAGAGAGAGAGAGAGTTTAG
L484_027201	GRAS	LOC21407115	122-141	-	3.77e-05	0.00423	GAGAGAGAGAGAGAGAGTTT
L484_005010	MYB	LOC21407115	16-30	+	8e-05	0.0241	TTGACAACCCCCAAC

>LOC21409963

Motif	Family	Seq ID	Position	Strand	p-value	q-value	Matched sequence
L484_024385	ARF	LOC21409963	70-90	-	1.56e-05	0.00529	TTTTTGAGTGTGGGAGGGAAA
L484_019276	BBR-BPC	LOC21409963	56-79	-	1.08e-05	0.00299	GGGAGGGAAAGAAGTGGAAGTGGA
L484_019400	BBR-BPC	LOC21409963	65-85	+	2.5e-05	0.00718	ACTTCTTTCCCTCCCACACTC
L484_019400	BBR-BPC	LOC21409963	61-81	+	5.09e-05	0.0073	TTCCACTTCTTTCCCTCCCAC
L484_019276	BBR-BPC	LOC21409963	58-81	-	6.36e-05	0.00882	GTGGGAGGGAAAGAAGTGGAAGTG
L484_025451	C2H2	LOC21409963	57-75	+	7.19e-06	0.00198	CCACTTCCACTTCTTTCCC
L484_003682	C2H2	LOC21409963	58-68	+	1.14e-05	0.00408	CACTTCCACTT
L484_025451	C2H2	LOC21409963	63-81	+	3.37e-05	0.00415	CCACTTCTTTCCCTCCCAC
L484_017006	C2H2	LOC21409963	75-85	-	3.96e-05	0.0138	GAGTGTGGGAG
L484_025451	C2H2	LOC21409963	60-78	+	4.53e-05	0.00415	CTTCCACTTCTTTCCCTCC
L484_003682	C2H2	LOC21409963	75-85	+	6.06e-05	0.0108	CTCCCACACTC
L484_017006	C2H2	LOC21409963	33-43	-	7.71e-05	0.0138	GAGTGAAATGA
L484_016920	Dof	LOC21409963	178-188	-	7.7e-05	0.0286	GAAAAAGGAAT
L484_012044	G2-like	LOC21409963	175-185	-	2.75e-06	0.000984	AAAGGAATGTT
L484_009894	G2-like	LOC21409963	171-185	+	5.54e-06	0.00197	CCCAAACATTCCTTT
L484_024949	GATA	LOC21409963	119-133	-	4.11e-06	0.00147	ATGGTTGGGATGATG
L484_007753	GATA	LOC21409963	116-134	+	6.98e-05	0.0249	TATCATCATCCCAACCATT
L484_011278	MYB	LOC21409963	121-139	+	2.7e-05	0.00867	TCATCCCAACCATTTTACC
L484_015941	MYB	LOC21409963	121-139	-	4.07e-05	0.0126	GGTAAAATGGTTGGGATGA
