# Supplementary material for: Transcriptional Regulation of the Phenylalanine Ammonia-Lyase (PAL) Gene Family in Mulberry Under Chitosan-Induced Stress
Source: Plants (Basel). 2025 Sep 5;14(17):2783. doi: 10.3390/plants14172783 (PMC12430538; doi:10.3390/plants14172783)
Supplement: Supplementary file 1 [file plants-14-02783-s001.zip › Table S2.pdf]

Table S2: List of TFs predicted by FootprintDB to bind to conserved sequence motifs A through C

This analysis employed a STAMP E-value threshold of 1e-03. Both this threshold and the DNA motif similarity scores were determined through BLASTP alignments against the 3D-footprint library. The calculations were based on the sum of the Pearson correlation coefficients of the aligned DNA motif positions, using data from the library accessible at floresta.eead.csic.es/3dfootprint/download/list_interface2dna.txt.

In the results presentation, rows are color-coded to indicate threshold compliance. Green-colored rows indicate sequences that successfully passed the twilight threshold.

The twilight threshold corresponds to specific identity and similarity thresholds. These were calculated for each sequence alignment by establishing cut-off values that progressively left 95%, 90%, and 75% of dissimilar TF-DNA complexes below the selected value, as detailed in Sebastian and Contreras-Moreira's 2013 publication (https://doi.org/10.1093/nar/gks1301).

For the footprintDB PWM / Consensus column interpretation, the display follows a specific format. The upper row shows the motif sequence within the PAL promoter, while the lower row displays the binding site of the TF candidate that footprintDB predicts could potentially bind to the motif. The matching bases between the motif and the TF binding site are represented in uppercase letters, while any mismatched bases are shown in lowercase.

The base letters presented follow the IUPAC nucleotide code:

IUPAC nucleotide code	Base
**A**	Adenine
**C**	Cytosine
**G**	Guanine
**T**	Thymine
**R**	A or G
**Y**	C or T
**S**	G or C
**W**	A or T
**K**	G or T
**M**	A or C
**B**	C or G or T
**D**	A or G or T
**H**	A or C or T
**V**	A or C or G
**N**	any base
**-**	gap

FootprintDB result of motif A
LOC21384641_motif A

Query: DNA TCCCTCTGTGGGGCCCAC

footprintDB template	Source	STAMP e-value	Motif similarity	footprinDB PWM / Consensus
MA1284.2: TCP1	JASPAR 2024	2.2e-16	10.80 / 11	TCCCTCTGTGGGGCCCAC<br>--------gkGGGrCCCAC
MA1287.2: TCP21	JASPAR 2024	2.2e-16	10.80 / 11	TCCCTCTGTGGGGCCCAC<br>--------gTGGGkCCCAC
MA1288.2: TCP22	JASPAR 2024	1.1e-15	10.64 / 11	TCCCTCTGTGGGGCCCAC<br>--------rTGGGkCCCAC
M0758: At2g45680.ampDAP / T08434;	AthalianaCistrome v4_May2016	1.3e-14	10.39 / 11	TCCCTCTGTGGGGCCCAC<br>--------gTGGGkCCCAC
M0757: At5g08330.ampDAP / T21230;	AthalianaCistrome v4_May2016	3.1e-14	10.70 / 11	TCCCTCTGTGGGGCCCAC-<br>--------rTgGGkCCCACw
M1649_1.02: AT5G51910 / T151061_1.02	CISBP 1.02	5.6e-14	9.77 / 10	TCCCTCTGTGGGGCCCAC<br>---------tgGGsCCCAC
MA1063.1: TCP19	JASPAR 2024	5.7e-14	9.77 / 10	TCCCTCTGTGGGGCCCAC<br>---------tgGGsCCCAC
M0759: T13995; / TCP20.ampDAP	AthalianaCistrome v4_May2016	1.5e-13	10.52 / 11	TCCCTCTGTGGGGCCCAC-<br>--------gTGGGkCCCACm
MA1291.1 / M0754: TCP7 / T23707; / TCP7.DAP	JASPAR 2024 AthalianaCistrome v4_May2016	2.6e-13	10.06 / 11	TCCCTCTGTGGGGCCCAC<br>--------GTGGGrCCCAy
MA1285.2: TCP9	JASPAR 2024	6.2e-13	9.97 / 11	TCCCTCTGTGGGGCCCAC<br>--------GTGGGaCCCAC

FootprintDB result of motif A
LOC24107112_motif A

Query: **DNA** **TCCCACCATGCACCCCAG**

footprintDB template	Source	STAMP e-value	Motif similarity	footprinDB PWM / Consensus
5yzy_C: B3 domain-containing transcription repressor VAL1	3D-footprint 20231221	3.4e-07	6.00 / 6	CTGGGGTGCATGGTGGGA<br>-------tGCATg------
6j9a_A: B3 domain-containing transcription repressor VAL1	3D-footprint 20231221	3.4e-07	6.00 / 6	CTGGGGTGCATGGTGGGA<br>------tGCATG------
5yzz_C: B3 domain-containing transcription repressor VAL1	3D-footprint 20231221	3.4e-07	6.00 / 6	CTGGGGTGCATGGTGGGA<br>------tGCATG------
6j9b_A: B3 domain-containing transcription factor FUS3	3D-footprint 20231221	3.7e-07	5.99 / 6	CTGGGGTGCATGGTGGGA<br>------tGCATg------
6j9c_D: B3 domain-containing transcription factor LEC2	3D-footprint 20231221	4.8e-07	5.96 / 6	CTGGGGTGCATGGTGGGA<br>------tGCATG------
MA0581.2: LEC2	JASPAR 2024	1.0e-06	5.87 / 6	CTGGGGTGCATGGTGGGA<br>------yGCATG------

FootprintDB result of motif A
LOC21407113_motif A

Query: DNA TCCCACCATGCACCCCAG

footprintDB template	Source	STAMP e-value	Motif similarity	footprinDB PWM / Consensus
5yzy_C: B3 domain-containing transcription repressor VAL1	3D-footprint 20231221	3.4e-07	6.00 / 6	CTGGGGTGCATGGTGGGA<br>-------tGCATg-------
6j9a_A: B3 domain-containing transcription repressor VAL1	3D-footprint 20231221	3.4e-07	6.00 / 6	CTGGGGTGCATGGTGGGA<br>-------tGCATG-------
5yzz_C: B3 domain-containing transcription repressor VAL1	3D-footprint 20231221	3.4e-07	6.00 / 6	CTGGGGTGCATGGTGGGA<br>-------tGCATG-------
6j9b_A: B3 domain-containing transcription factor FUS3	3D-footprint 20231221	3.7e-07	5.99 / 6	CTGGGGTGCATGGTGGGA<br>-------tGCATg-------
6j9c_D: B3 domain-containing transcription factor LEC2	3D-footprint 20231221	4.8e-07	5.96 / 6	CTGGGGTGCATGGTGGGA<br>-------tGCATG-------
MA0581.2: LEC2	JASPAR 2024	1.0e-06	5.87 / 6	CTGGGGTGCATGGTGGGA<br>-------yGCATG-------

FootprintDB result of motif A
LOC21407114_motif A

Query: DNA TCACACACTGCCCCACAC

footprintDB template	Source	STAMP e-value	Motif similarity	footprinDB PWM / Consensus
EEAD0078: CORE	EEADannot 2023-12-22	4.4e-05	5.00 / 5	GTGTGGGGCAGTGTGTGA<br>-------------TGTGA
UN0842.1: ZAT7	JASPAR 2024	7.5e-05	4.93 / 5	GTGTGGGGCAGTGTGTGA<br>---------AGTGt----
MA1063.2: TCP19	JASPAR 2024	1.1e-04	6.15 / 8	GTGTGGGGCAGTGTGTGA<br>--GTGGGsCC--------
UN0839.1: TRFL5	JASPAR 2024	2.3e-04	5.60 / 7	GTGTGGGGCAGTGTGTGA<br>---TAGGGCa--------

FootprintDB result of motif A
LOC21407115_motif A

Query: DNA TCCCACCATGCACCCCAG

footprintDB template	Source	STAMP e-value	Motif similarity	footprinDB PWM / Consensus
5yzy_C: B3 domain-containing transcription repressor VAL1	3D-footprint 20231221	3.4e-07	6.00 / 6	CTGGGGTGCATGGTGGGA<br>-------tGCATg------
6j9a_A: B3 domain-containing transcription repressor VAL1	3D-footprint 20231221	3.4e-07	6.00 / 6	CTGGGGTGCATGGTGGGA<br>-------tGCATG------
5yzz_C: B3 domain-containing transcription repressor VAL1	3D-footprint 20231221	3.4e-07	6.00 / 6	CTGGGGTGCATGGTGGGA<br>-------tGCATG------
6j9b_A: B3 domain-containing transcription factor FUS3	3D-footprint 20231221	3.7e-07	5.99 / 6	CTGGGGTGCATGGTGGGA<br>-------tGCATg------
6j9c_D: B3 domain-containing transcription factor LEC2	3D-footprint 20231221	4.8e-07	5.96 / 6	CTGGGGTGCATGGTGGGA<br>-------tGCATG------
MA0581.2: LEC2	JASPAR 2024	1.0e-06	5.87 / 6	CTGGGGTGCATGGTGGGA<br>-------yGCATG------

FootprintDB result of motif A
LOC21409963_motif A

Query: DNA TTCCCACATGCACCACAC

footprintDB template	Source	STAMP e-value	Motif similarity	footprinDB PWM / Consensus
MA0565.3: FUS3	JASPAR 2024	7.3e-09	7.00 / 7	GTGTGGTGCATGTGGGAA<br>-------TGCATGT-----
M0162_1.02: SPT / T012466_1.02	CISBP 1.02	1.4e-07	7.63 / 9	GTGTGGTGCATGTGGGAA<br>--------gCACGTGsg--
MA1061.1: SPT	JASPAR 2024	1.4e-07	7.63 / 9	GTGTGGTGCATGTGGGAA<br>--------gCACGTGsg--
5yzy_C: B3 domain-containing transcription repressor VAL1	3D-footprint 20231221	3.4e-07	6.00 / 6	GTGTGGTGCATGTGGGAA<br>-------tGCATg------
6j9a_A: B3 domain-containing transcription repressor VAL1	3D-footprint 20231221	3.4e-07	6.00 / 6	GTGTGGTGCATGTGGGAA<br>-------tGCATG------
5yzz_C: B3 domain-containing transcription repressor VAL1	3D-footprint 20231221	3.4e-07	6.00 / 6	GTGTGGTGCATGTGGGAA<br>-------tGCATG------
6j9b_A: B3 domain-containing transcription factor FUS3	3D-footprint 20231221	3.7e-07	5.99 / 6	GTGTGGTGCATGTGGGAA<br>-------tGCATg------
6j9c_D: B3 domain-containing transcription factor LEC2	3D-footprint 20231221	4.8e-07	5.96 / 6	GTGTGGTGCATGTGGGAA<br>-------tGCATG------
MA0581.2: LEC2	JASPAR 2024	1.0e-06	5.87 / 6	GTGTGGTGCATGTGGGAA<br>-------yGCATG------

FootprintDB result of motif B
LOC21384641_Motif B

Query: DNA CACGCAAACCAAAGCA

footprintDB template	Source	STAMP e-value	Motif similarity	footprinDB PWM / Consensus
M1405_1.02: ANAC079 / T127990_1.02	CISBP 1.02	6.5e-07	6.87 / 7	TGCTTTGGTTTGCGTG-<br>---------TTGCGTRt
MA0939.1: NAC079 / NAC080	JASPAR 2024	6.5e-07	6.87 / 7	TGCTTTGGTTTGCGTG-<br>---------TTGCGTRt
MA0938.1: NAC058	JASPAR 2024	7.4e-07	6.85 / 7	TGCTTTGGTTTGCGTG-<br>---------TtGCKTgy
MA1415.1: REF6	JASPAR 2024	1.7e-06	7.87 / 11	TGCTTTGGTTTGCGTG<br>trCTCTGTTTy-----
MA0936.1: NAC046 / T11I18.17	JASPAR 2024	5.3e-06	6.57 / 7	TGCTTTGGTTTGCGTG-<br>---------TkgCGTrt
M1408_1.02: ANAC100 / T128011_1.02	CISBP 1.02	1.7e-05	7.11 / 9	TGCTTTGGTTTGCGTG-<br>-------grtwrckTry
MA1043.2: NAC083	JASPAR 2024	2.0e-05	5.95 / 7	TGCTTTGGTTTGCGTG<br>---------TTrCGTr
UP00576A_1: NTL6 / transcription factor NTL6	UniPROBE 20160601	2.3e-05	7.68 / 9	TGCTTTGGTTTGCGTG-<br>--<br>-------GgTTGCGTGTas
UP00572A_3: ANAC092 / ARABIDOPSIS NAC DOMAIN CONTAINING PROTEIN 2 / ARABIDOPSIS NAC DOMAIN CONTAINING PROTEIN 6 / Arabidopsis NAC domain containing protein 92 / ATNAC2 / MIJ24_11 / MIJ24.11 / NAC-domain transcription factor / NAC6 / ORE1 / ORESARA 1	UniPROBE 20160601	2.5e-05	7.66 / 9	TGCTTTGGTTTGCGTG-<br>--<br>-------GGTTGCGTGTwg

FootprintDB result of motif B
LOC24107112_Motif B

Query: DNA CAGCCAAATCACAGCC

footprintDB template	Source	STAMP e-value	Motif similarity	footprinDB PWM / Consensus
EEAD0078: CORE	EEADannot 2023-12-22	3.3e-05	5.00 / 5	GGCTGTGATTTGGCTG<br>---TGTGA--------
M0464: T08131; / WLIM2A.DAP	AthalianaCistrome v4_May2016	4.9e-05	5.81 / 7	GGCTGTGATTTGGCTG<br>------sATTTGA---
UN0862.1: HAT3	JASPAR 2024	3.9e-04	4.99 / 6	GGCTGTGATTTGGCTG<br>-----TGATTr-----

FootprintDB result of motif B
LOC21407113_Motif B

Query: DNA CAGCCAAATCACAGCC

footprintDB template	Source	STAMP e-value	Motif similarity	footprinDB PWM / Consensus
EEAD0078: CORE	EEADannot 2023-12-22	3.3e-05	5.00 / 5	GGCTGTGATTTGGCTG<br>---TGTGA--------
M0464: T08131; / WLIM2A.DAP	AthalianaCistrome v4_May2016	4.9e-05	5.81 / 7	GGCTGTGATTTGGCTG<br>-------sATTTGA---
UN0862.1: HAT3	JASPAR 2024	3.9e-04	4.99 / 6	GGCTGTGATTTGGCTG<br>-----TGATTr-----

FootprintDB result of motif B
LOC21407114_Motif B

Query: DNA CAGCCAAAAGAAGGCC

footprintDB template	Source	STAMP e-value	Motif similarity	footprinDB PWM / Consensus
MA0563.2: SEP3	JASPAR 2024	1.4e-06	7.53 / 10	CAGCCAAAAGAAGGCC<br>---CCAAAAAwrG---
MA1272.3: DOF2.2	JASPAR 2024	3.7e-04	5.00 / 6	CAGCCAAAAGAAGGCC<br>-----AAAAAG------

FootprintDB result of motif B
LOC21407115_Motif B

Query: DNA CAGCCAAATCACAGCC

footprintDB template	Source	STAMP e-value	Motif similarity	footprinDB PWM / Consensus
EEAD0078: CORE	EEADannot 2023-12-22	3.3e-05	5.00 / 5	**GGCTGTGATTTGGCTG**<br>**---TGTGA---------**
M0464: T08131; / WLIM2A.DAP	AthalianaCistrome v4_May2016	4.9e-05	5.81 / 7	**GGCTGTGATTTGGCTG**<br>**-------sATTTGA---**
UN0862.1: HAT3	JASPAR 2024	3.9e-04	4.99 / 6	**GGCTGTGATTTGGCTG**<br>**------TGATTr------**

FootprintDB result of motif B
LOC21409963_Motif B

Query: DNA CCGCCAGAGCTCAGCA

footprintDB template	Source	STAMP e-value	Motif similarity	footprinDB PWM / Consensus
MA0579.2: CDC5	JASPAR 2024	9.7e-06	6.89 / 8	**-TGCTGAGCTCTGGCGG**<br>**sCGCTGAGs--------**
5wx9_A: Ethylene-responsive transcription factor ERF096	3D-footprint 20231221	3.2e-05	7.00 / 7	**TGCTGAGCTCTGGCGG---**<br>**---------CTGGcGGCTa**
ERF1 / ERF2 / ERF5: ERF1 / ERF2 / ERF5	Athamap 20091028	1.6e-04	6.00 / 6	**TGCTGAGCTCTGGCGG--**<br>**----------TGGCGGCT**
MA0567.1: ERF1B	JASPAR 2024	2.2e-04	5.94 / 6	**TGCTGAGCTCTGGCGG--**<br>**----------tGGCGGCk**
MA1004.1: ERF13	JASPAR 2024	3.1e-04	5.88 / 6	**TGCTGAGCTCTGGCGG--**<br>**----------yGgCGGCs**

FootprintDB result of motif C
LOC21384641_Motif C

Query: DNA CTAGCACTCTATGTAGCT

footprintDB template	Source	STAMP e-value	Motif similarity	footprinDB PWM / Consensus
MA1797.1: RAV2	JASPAR 2024	1.6e-05	6.46 / 8	CTAGCACTCTATGTAGCT<br>---------tATGTTGC-
M0013_1.02: T000607_1.02 / TEM2	CISBP 1.02	1.6e-05	6.46 / 8	CTAGCACTCTATGTAGCT<br>---------kATGTTGC-
MA1800.1: TEM1	JASPAR 2024	2.4e-05	6.40 / 8	CTAGCACTCTATGTAGCT<br>---------wATGTTGC-
M0008_1.02: T000590_1.02 / TEM1	CISBP 1.02	2.4e-05	6.40 / 8	CTAGCACTCTATGTAGCT<br>---------wATGTTGC-
MA1381.2: AT3G46070	JASPAR 2024	3.4e-05	5.91 / 7	CTAGCACTCTATGTAGCT<br>---TCACTct--------
UN0397.1: EDF3	JASPAR 2024	6.0e-05	6.25 / 8	CTAGCACTCTATGTAGCT<br>---------TmTGTTGy-
M0081_1.02: EDF3 / T004631_1.02	CISBP 1.02	6.0e-05	6.25 / 8	CTAGCACTCTATGTAGCT<br>---------TmTGTTGy-
UN0407.2: REM1	JASPAR 2024	3.1e-04	5.54 / 7	CTAGCACTCTATGTAGCT<br>---------GwTGTAG--

FootprintDB result of motif C
LOC24107112_Motif C

Query: DNA CTCTCCCTCTCTCTAGCT

footprintDB template	Source	STAMP e-value	Motif similarity	footprinDB PWM / Consensus
MA1402.1 / M0151: BPC6 / BPC6.DAP / T22052;	JASPAR 2024<br>AthalianaCistrome v4_May2016	0	14.00 / 18	-AGCTAGAGAGAGGGAGAG--<br>kAGAGAGAGAGAGAGAGAGAG
MA1403.1 / M0152: BPC5 / BPC5.DAP / T20837;	JASPAR 2024<br>AthalianaCistrome v4_May2016	0	14.01 / 18	--AGCTAGAGAGAGGGAGAG----------<br>AGAGAGAGAGAGAGAGAGAGAGAGAGAGAG
MA1404.1 / M0150: BPC1 / BPC1.DAP / T10218;	JASPAR 2024<br>AthalianaCistrome v4_May2016	0	13.99 / 18	---AGCTAGAGAGAGGGAGAG---<br>GAGAGAGAGAGAGAGAGAGAGAGA
M0713: FRS9.DAP / T19961;	AthalianaCistrome v4_May2016	0	13.82 / 18	---AGCTAGAGAGAGGGAGAG<br>GAGAGAGAGAGAGAGAGAGAG
M0149: BPC1.ampDAP / T10218;	AthalianaCistrome v4_May2016	1.1e-16	12.52 / 15	AGCTAGAGAGAGGGAGAG<br>---rAGAGAGAGArAGAg
M0153: BPC5.ampDAP / T20837;	AthalianaCistrome v4_May2016	1.1e-16	13.93 / 18	-----------AGCTAGAGAGAGGGAGAG<br>rrraraaraGAGARAGAGAGAGAGAGAGAG
MA1381.2: AT3G46070	JASPAR 2024	2.5e-04	5.58 / 7	AGCTAGAGAGAGGGAGAG<br>----agAGTGA-------

FootprintDB result of motif C
LOC21407113_Motif C

Query: DNA CTCTCTCTCTCTCTAGCT

footprintDB template	Source	STAMP e-value	Motif similarity	footprinDB PWM / Consensus
MA1402.1 / M0151: BPC6 / BPC6.DAP / T22052;	JASPAR 2024 AthalianaCistrome v4_May2016	0	15.31 / 18	-AGCTAGAGAGAGAGAGAG--<br>kAGAGAGAGAGAGAGAGAGAG
MA1403.1 / M0152: BPC5 / BPC5.DAP / T20837;	JASPAR 2024 AthalianaCistrome v4_May2016	0	15.34 / 18	AGCTAGAGAGAGAGAGAG------------<br>AGAGAGAGAGAGAGAGAGAGAGAGAGAGAG
MA1404.1 / M0150: BPC1 / BPC1.DAP / T10218;	JASPAR 2024 AthalianaCistrome v4_May2016	0	15.23 / 18	-----AGCTAGAGAGAGAGAGAG-<br>GAGAGAGAGAGAGAGAGAGAGAGA
M0149: BPC1.ampDAP / T10218;	AthalianaCistrome v4_May2016	0	13.83 / 15	AGCTAGAGAGAGAGAG<br>---rAGAGAGAGArAGAg
M0153: BPC5.ampDAP / T20837;	AthalianaCistrome v4_May2016	0	15.13 / 18	-----------AGCTAGAGAGAGAGAGAG<br>rrraraaraGAGARAGAGAGAGAGAGAG
M0713: FRS9.DAP / T19961;	AthalianaCistrome v4_May2016	0	15.10 / 18	---AGCTAGAGAGAGAGAGAG<br>GAGAGAGAGAGAGAGAGAGAG

FootprintDB result of motif C
LOC21407114_Motif C

Query: DNA CCAACCCTGTCACTAGCC

footprintDB template	Source	STAMP e-value	Motif similarity	footprinDB PWM / Consensus
M0855_1.02: KNAT3 / T090264_1.02	CISBP 1.02	1.0e-05	7.32 / 10	CCAACCCTGTCACTAGCC<br>----asmtGTCahw----
MA1736.2: AT5G04390	JASPAR 2024	1.1e-04	4.88 / 5	CCAACCCTGTCACTAGCC<br>---------wCACT----
6r2v_A: Nuclear transcription factor Y subunit A-6	3D-footprint 20231221	1.6e-04	5.66 / 7	CCAACCCTGTCACTAGCC<br>CCAATcc-----------
	AthaMYB 1.0	2.5e-04	6.00 / 6	--<br>CCAACCCTGTCACTAGCC<br>CACCAACC------------

FootprintDB result of motif C
LOC21407115_Motif C

Query: DNA CTCTCTCTCTCTCTAGCT

footprintDB template	Source	STAMP e-value	Motif similarity	footprinDB PWM / Consensus
MA1402.1 / M0151: BPC6 / BPC6.DAP / T22052;	JASPAR 2024 AthalianaCistrome v4_May2016	0	15.31 / 18	-AGCTAGAGAGAGAGAGAG--<br>kAGAGAGAGAGAGAGAGAGAG
MA1403.1 / M0152: BPC5 / BPC5.DAP / T20837;	JASPAR 2024 AthalianaCistrome v4_May2016	0	15.34 / 18	AGCTAGAGAGAGAGAGAG------------<br>AGAGAGAGAGAGAGAGAGAGAGAGAGAGAG
MA1404.1 / M0150: BPC1 / BPC1.DAP / T10218;	JASPAR 2024 AthalianaCistrome v4_May2016	0	15.23 / 18	-----AGCTAGAGAGAGAGAGAG-<br>GAGAGAGAGAGAGAGAGAGAGAGA
M0149: BPC1.ampDAP / T10218;	AthalianaCistrome v4_May2016	0	13.83 / 15	AGCTAGAGAGAGAGAG<br>---rAGAGAGAGArAGAg
M0153: BPC5.ampDAP / T20837;	AthalianaCistrome v4_May2016	0	15.13 / 18	-----------AGCTAGAGAGAGAGAGAG<br>rrraraaraGAGARAGAGAGAGAGAGAG
M0713: FRS9.DAP / T19961;	AthalianaCistrome v4_May2016	0	15.10 / 18	---AGCTAGAGAGAGAGAGAG<br>GAGAGAGAGAGAGAGAGAGAG

FootprintDB result of motif C
LOC21409963_Motif C

Query: DNA CTCTTGCTCTCGCAAGCC

footprintDB template	Source	STAMP e-value	Motif similarity	footprinDB PWM / Consensus
M0007: AT5G25475.DAP / T27115;	AthalianaCistrome v4_May2016	5.3e-04	4.99 / 6	GGCTTGCGAGAGCAAGAG TGCTTG------------
UN0848.1: AT5G25475	JASPAR 2024	5.3e-04	4.99 / 6	GGCTTGCGAGAGCAAGAG TGCTTG------------
